# *N*-(1*H*-Indazol-5-yl)-4-meth­oxy­benzene­sulfonamide

**DOI:** 10.1107/S1600536813028912

**Published:** 2013-10-26

**Authors:** Hakima Chicha, El Mostapha Rakib, Latifa Bouissane, Mohamed Saadi, Lahcen El Ammari

**Affiliations:** a Laboratoire de Chimie Organique et Analytique, Université Sultan Moulay Slimane, Faculté des Sciences et Techniques, Béni-Mellal, BP 523, Morocco; b Laboratoire de Chimie du Solide Appliquée, Faculté des Sciences, Université Mohammed V-Agdal, Avenue Ibn Battouta, BP 1014, Rabat, Morocco

## Abstract

In the title compound, C_14_H_13_N_3_O_3_S, the fused ring system is almost planar, the largest deviation from the mean plane being 0.023 (2) Å, and makes a dihedral angle of 47.92 (10)° with the benzene ring of the benzene­sulfonamide moiety. In the crystal, mol­ecules are connected through N—H⋯O hydrogen bonds and weak C—H⋯O contacts, forming a two-dimensional network which is parallel to (010).

## Related literature

For the pharmacological activity of selected sulfonamide derivatives, see: El-Sayed *et al.* (2011 ▶); Smith & Jones (2008 ▶); Scozzafava *et al.* (2003 ▶). For similar compounds, see: Bouissane *et al.* (2006 ▶); Abbassi *et al.* (2012 ▶, 2013 ▶); Chicha *et al.* (2013 ▶).
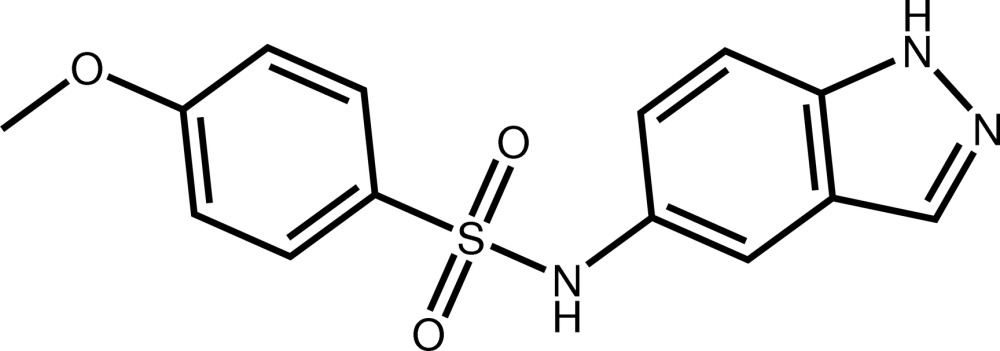


## Experimental

### 

#### Crystal data

C_14_H_13_N_3_O_3_S
*M* *_r_* = 303.33
Monoclinic, 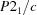

*a* = 8.9996 (4) Å
*b* = 7.1999 (3) Å
*c* = 21.3728 (10) Å
β = 91.794 (3)°
*V* = 1384.20 (11) Å^3^
*Z* = 4
Mo *K*α radiation
μ = 0.25 mm^−1^
*T* = 296 K
0.41 × 0.36 × 0.27 mm

#### Data collection

Bruker X8 APEX diffractometer
Absorption correction: multi-scan (*SADABS*; Bruker, 2009 ▶) *T*_min_ = 0.912, *T*_max_ = 0.954
14415 measured reflections
3059 independent reflections
2234 reflections with *I* > 2σ(*I*)
*R*_int_ = 0.039

#### Refinement

*R*[*F* ^2^ > 2σ(*F* ^2^)] = 0.042
*wR*(*F* ^2^) = 0.119
*S* = 1.05
3059 reflections
192 parameters
H-atom parameters constrained
Δρ_max_ = 0.26 e Å^−3^
Δρ_min_ = −0.32 e Å^−3^

### 

Data collection: *APEX2* (Bruker, 2009 ▶); cell refinement: *SAINT* (Bruker, 2009 ▶); data reduction: *SAINT*; program(s) used to solve structure: *SHELXS97* (Sheldrick, 2008 ▶); program(s) used to refine structure: *SHELXL97* (Sheldrick, 2008 ▶); molecular graphics: *ORTEP-3 for Windows* (Farrugia, 2012 ▶); software used to prepare material for publication: *PLATON* (Spek, 2009 ▶) and *publCIF* (Westrip, 2010 ▶).

## Supplementary Material

Crystal structure: contains datablock(s) I. DOI: 10.1107/S1600536813028912/im2443sup1.cif

Structure factors: contains datablock(s) I. DOI: 10.1107/S1600536813028912/im2443Isup2.hkl

Supplementary material file. DOI: 10.1107/S1600536813028912/im2443Isup3.cml

Additional supplementary materials:  crystallographic information; 3D view; checkCIF report

## Figures and Tables

**Table 1:** Hydrogen-bond geometry (Å, °)

*D*—H⋯*A*	*D*—H	H⋯*A*	*D*⋯*A*	*D*—H⋯*A*
N1—H1*N*⋯O1^i^	0.90	2.07	2.956 (2)	168
N3—H3*N*⋯O2^ii^	0.77	2.23	2.998 (2)	172
C6—H6⋯O2^i^	0.93	2.52	3.381 (2)	155

Symmetry codes: (i) 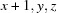; (ii) 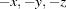.

**Fig. 1.**
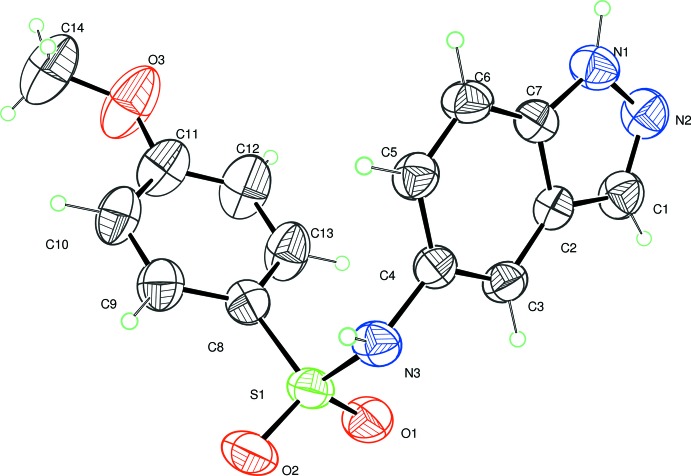
Molecular structure of the title compound showing displacement ellipsoids at the 50% probability level. H atoms are represented as small circles.

**Fig. 2.**
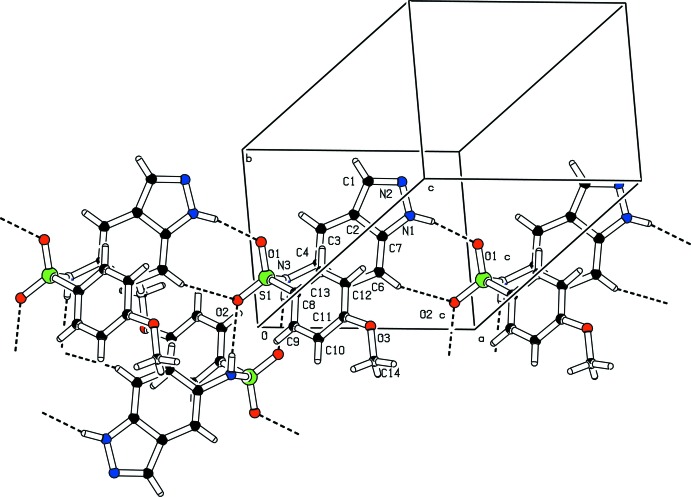
Partial crystal packing for the title compound showing N1–H1N···O1, N3–H3N···O2 and C6–H6···O2 hydrogen bonds as dashed lines.

## supplementary crystallographic information

### 1. Comment

Sulfonamides are an important class of compounds which are widely used in the design of diverse classes of drug candidates (El-Sayed *et al.*, 2011; Smith & Jones, 2008; Scozzafava *et al.*, 2003). Recently, some *N*-[7(6)-indazolyl]arylsulfonamides prepared by our research group showed important antiproliferative activity against some human and murine cell lines (Abbassi *et al.*, 2012, 2013; Bouissane *et al.*, 2006; Chicha *et al.*, 2013).

The molecule of the title compound is built up from two fused five- and six-membered rings (N1/N2/C1 to C7) linked to the 4-methoxybenzenesulfonamide group, as shown in Fig. 1. The fused ring system is almost planar, with the maximum deviation of -0.023 (2) Å arising from atom C1. Moreover, the dihedral angle between the indazole system and the plan through the atoms forming the benzene ring (C8 to C13) is 47.92 (10)°.

In the crystal, molecules are connected through N—H···O hydrogen bonds and weak C—H···O contacts, forming a two-dimensional network nearly parallel to (0 1 0) as shown in Fig.2 and Table 1.

### 2. Experimental

A mixture of 5-nitroindazole (216 mg, 1.22 mmol) and anhydrous SnCl_2_ (1.1 g, 6.1 mmol) in 25 ml of absolute ethanol was heated at 333 K for 6 h. After reduction, the starting material disappeared and the solution was allowed to cool down. The pH was made slightly basic (pH 7–8) by addition of 5% aqueous potassium bicarbonate before extraction with ethyl acetate. The organic phase was washed with brine and dried over magnesium sulfate. The solvent was removed to afford the amine, which was immediately dissolved in pyridine (5 ml) and then reacted with 4-methoxybenzenesulfonyl chloride (1.25 mmol) at room temperature for 24 h. After the reaction mixture was concentrated *in vacuo*, the resulting residue was purified by flash chromatography (eluted with ethyl acetate: hexane 2:8, yield: 54%, m.p.: 437 K). The title compound was recrystallized from ethanol.

### 3. Refinement

H atoms were located in a difference map and treated as riding with C–H = 0.96 Å, C–H = 0.93 Å, and N–H = 0.89 Å for methyl, aromatic CH and NH, respectively. Thermal parameters for hydrogen atoms were refined as *U*_iso_(H) = 1.2 *U*_eq_(C) (aromatic CH, NH) and *U*_iso_(H) = 1.5 *U*_eq_(C) for the methyl group.

### Crystal data

**Table:**

C_14_H_13_N_3_O_3_S	*F*(000) = 632
*M**_r_* = 303.33	*D*_x_ = 1.456 Mg m^−^^3^
Monoclinic, *P*2_1_/*c*	Mo *K*α radiation, λ = 0.71073 Å
Hall symbol: -P 2ybc	Cell parameters from 3059 reflections
*a* = 8.9996 (4) Å	θ = 2.3–27.1°
*b* = 7.1999 (3) Å	µ = 0.25 mm^−^^1^
*c* = 21.3728 (10) Å	*T* = 296 K
β = 91.794 (3)°	Block, colourless
*V* = 1384.20 (11) Å^3^	0.41 × 0.36 × 0.27 mm
*Z* = 4	

### Data collection

**Table:**

Bruker X8 APEX diffractometer	3059 independent reflections
Radiation source: fine-focus sealed tube	2234 reflections with *I* > 2σ(*I*)
Graphite monochromator	*R*_int_ = 0.039
φ and ω scans	θ_max_ = 27.1°, θ_min_ = 2.3°
Absorption correction: multi-scan (*SADABS*; Bruker, 2009)	*h* = −11→11
*T*_min_ = 0.912, *T*_max_ = 0.954	*k* = −9→7
14415 measured reflections	*l* = −26→27

### Refinement

**Table:**

Refinement on *F*^2^	Primary atom site location: structure-invariant direct methods
Least-squares matrix: full	Secondary atom site location: difference Fourier map
*R*[*F*^2^ > 2σ(*F*^2^)] = 0.042	Hydrogen site location: difference Fourier map
*wR*(*F*^2^) = 0.119	H-atom parameters constrained
*S* = 1.05	*w* = 1/[σ^2^(*F*_o_^2^) + (0.0542*P*)^2^ + 0.3443*P*] where *P* = (*F*_o_^2^ + 2*F*_c_^2^)/3
3059 reflections	(Δ/σ)_max_ < 0.001
192 parameters	Δρ_max_ = 0.26 e Å^−^^3^
0 restraints	Δρ_min_ = −0.32 e Å^−^^3^

### Special details

**Table:**

Geometry. All s.u.'s (except the s.u. in the dihedral angle between two l.s. planes) are estimated using the full covariance matrix. The cell s.u.'s are taken into account individually in the estimation of s.u.'s in distances, angles and torsion angles; correlations between s.u.'s in cell parameters are only used when they are defined by crystal symmetry. An approximate (isotropic) treatment of cell s.u.'s is used for estimating s.u.'s involving l.s. planes.
Refinement. Refinement of *F*^2^ against all reflections. The weighted *R*-factor *wR* and goodness of fit *S* are based on *F*^2^, conventional *R*-factors *R* are based on *F*, with *F* set to zero for negative *F*^2^. The threshold expression of *F*^2^ > 2σ(*F*^2^) is used only for calculating *R*-factors(gt) *etc*. and is not relevant to the choice of reflections for refinement. *R*-factors based on *F*^2^ are statistically about twice as large as those based on *F*, and *R*- factors based on all data will be even larger.

### Fractional atomic coordinates and isotropic or equivalent isotropic displacement parameters (Å^2^)

**Table:**

	*x*	*y*	*z*	*U*_iso_*/*U*_eq_	
C1	0.4773 (2)	0.7356 (3)	0.10971 (11)	0.0511 (5)	
H1	0.4212	0.8416	0.1167	0.061*	
C2	0.4174 (2)	0.5666 (3)	0.08594 (9)	0.0393 (4)	
C3	0.2770 (2)	0.5028 (3)	0.06477 (9)	0.0418 (5)	
H3	0.1947	0.5810	0.0638	0.050*	
C4	0.26542 (19)	0.3211 (3)	0.04565 (9)	0.0375 (4)	
C5	0.3905 (2)	0.2042 (3)	0.04520 (10)	0.0432 (5)	
H5	0.3786	0.0819	0.0320	0.052*	
C6	0.5286 (2)	0.2648 (3)	0.06360 (10)	0.0468 (5)	
H6	0.6112	0.1874	0.0624	0.056*	
C7	0.5405 (2)	0.4478 (3)	0.08428 (9)	0.0399 (4)	
C8	0.0730 (2)	0.0780 (3)	0.13810 (10)	0.0443 (5)	
C9	0.0520 (3)	−0.1113 (3)	0.13918 (12)	0.0564 (6)	
H9	−0.0086	−0.1682	0.1088	0.068*	
C10	0.1216 (3)	−0.2164 (3)	0.18576 (12)	0.0631 (6)	
H10	0.1068	−0.3442	0.1872	0.076*	
C11	0.2130 (3)	−0.1310 (4)	0.23000 (11)	0.0658 (7)	
C12	0.2318 (4)	0.0582 (4)	0.22939 (12)	0.0789 (8)	
H12	0.2913	0.1154	0.2601	0.095*	
C13	0.1630 (3)	0.1618 (3)	0.18373 (11)	0.0649 (7)	
H13	0.1765	0.2899	0.1831	0.078*	
C14	0.2942 (4)	−0.4178 (4)	0.27574 (15)	0.0991 (11)	
H14A	0.3528	−0.4620	0.3110	0.149*	
H14B	0.3375	−0.4594	0.2377	0.149*	
H14C	0.1948	−0.4653	0.2779	0.149*	
N1	0.65971 (18)	0.5491 (3)	0.10488 (8)	0.0496 (4)	
H1N	0.7553	0.5150	0.1082	0.073 (8)*	
N2	0.6220 (2)	0.7245 (3)	0.12066 (9)	0.0543 (5)	
N3	0.12368 (17)	0.2461 (2)	0.02574 (8)	0.0442 (4)	
H3N	0.1279	0.1587	0.0047	0.049 (7)*	
O1	−0.03673 (15)	0.3928 (2)	0.10350 (8)	0.0574 (4)	
O2	−0.11991 (14)	0.1127 (2)	0.04547 (8)	0.0589 (4)	
O3	0.2906 (3)	−0.2221 (3)	0.27662 (9)	0.1019 (8)	
S1	−0.00356 (5)	0.21505 (7)	0.07747 (3)	0.04454 (17)	

### Atomic displacement parameters (Å^2^)

**Table:**

	*U*^11^	*U*^22^	*U*^33^	*U*^12^	*U*^13^	*U*^23^
C1	0.0589 (13)	0.0394 (11)	0.0548 (14)	−0.0011 (10)	−0.0028 (10)	−0.0053 (10)
C2	0.0456 (10)	0.0356 (10)	0.0365 (10)	0.0006 (8)	0.0010 (8)	0.0011 (8)
C3	0.0415 (10)	0.0377 (11)	0.0459 (11)	0.0054 (8)	−0.0026 (8)	0.0007 (9)
C4	0.0389 (9)	0.0398 (11)	0.0339 (10)	−0.0002 (8)	−0.0002 (7)	0.0013 (8)
C5	0.0469 (10)	0.0358 (10)	0.0468 (12)	0.0034 (8)	0.0020 (9)	−0.0047 (9)
C6	0.0400 (10)	0.0464 (12)	0.0542 (13)	0.0079 (9)	0.0020 (9)	−0.0065 (10)
C7	0.0389 (9)	0.0454 (11)	0.0354 (10)	−0.0010 (8)	0.0009 (8)	−0.0005 (8)
C8	0.0443 (10)	0.0438 (12)	0.0451 (12)	0.0001 (9)	0.0055 (9)	−0.0046 (9)
C9	0.0622 (13)	0.0440 (13)	0.0630 (15)	−0.0017 (10)	−0.0010 (11)	−0.0081 (11)
C10	0.0843 (17)	0.0379 (12)	0.0674 (17)	0.0027 (12)	0.0079 (13)	−0.0046 (11)
C11	0.0959 (18)	0.0586 (16)	0.0429 (14)	0.0103 (14)	0.0008 (12)	−0.0005 (11)
C12	0.123 (2)	0.0618 (17)	0.0505 (15)	−0.0090 (16)	−0.0227 (15)	−0.0034 (12)
C13	0.0962 (18)	0.0471 (13)	0.0505 (14)	−0.0087 (13)	−0.0114 (13)	−0.0016 (11)
C14	0.154 (3)	0.0627 (19)	0.080 (2)	0.0185 (19)	−0.006 (2)	0.0124 (16)
N1	0.0429 (9)	0.0535 (11)	0.0522 (11)	−0.0027 (8)	−0.0032 (8)	−0.0073 (8)
N2	0.0598 (11)	0.0472 (11)	0.0554 (11)	−0.0070 (9)	−0.0059 (9)	−0.0075 (9)
N3	0.0425 (9)	0.0444 (10)	0.0452 (10)	−0.0006 (7)	−0.0059 (7)	−0.0074 (8)
O1	0.0471 (8)	0.0472 (9)	0.0780 (11)	0.0083 (7)	0.0036 (7)	−0.0120 (8)
O2	0.0357 (7)	0.0605 (10)	0.0797 (11)	−0.0009 (7)	−0.0091 (7)	−0.0128 (8)
O3	0.179 (2)	0.0620 (12)	0.0623 (13)	0.0153 (13)	−0.0334 (14)	0.0025 (10)
S1	0.0340 (2)	0.0421 (3)	0.0572 (3)	0.0021 (2)	−0.0027 (2)	−0.0070 (2)

### Geometric parameters (Å, º)

**Table:**

C1—N2	1.319 (3)	C9—H9	0.9300
C1—C2	1.418 (3)	C10—C11	1.378 (3)
C1—H1	0.9300	C10—H10	0.9300
C2—C7	1.401 (3)	C11—O3	1.366 (3)
C2—C3	1.406 (3)	C11—C12	1.373 (4)
C3—C4	1.374 (3)	C12—C13	1.362 (3)
C3—H3	0.9300	C12—H12	0.9300
C4—C5	1.405 (3)	C13—H13	0.9300
C4—N3	1.437 (2)	C14—O3	1.410 (3)
C5—C6	1.364 (3)	C14—H14A	0.9600
C5—H5	0.9300	C14—H14B	0.9600
C6—C7	1.393 (3)	C14—H14C	0.9600
C6—H6	0.9300	N1—N2	1.353 (2)
C7—N1	1.359 (2)	N1—H1N	0.8951
C8—C9	1.376 (3)	N3—S1	1.6319 (18)
C8—C13	1.387 (3)	N3—H3N	0.7745
C8—S1	1.753 (2)	O1—S1	1.4306 (15)
C9—C10	1.385 (3)	O2—S1	1.4361 (14)
			
N2—C1—C2	111.95 (19)	O3—C11—C12	115.0 (2)
N2—C1—H1	124.0	O3—C11—C10	124.5 (2)
C2—C1—H1	124.0	C12—C11—C10	120.5 (2)
C7—C2—C3	119.72 (17)	C13—C12—C11	119.8 (2)
C7—C2—C1	103.96 (17)	C13—C12—H12	120.1
C3—C2—C1	136.31 (19)	C11—C12—H12	120.1
C4—C3—C2	117.75 (17)	C12—C13—C8	120.5 (2)
C4—C3—H3	121.1	C12—C13—H13	119.7
C2—C3—H3	121.1	C8—C13—H13	119.7
C3—C4—C5	121.28 (17)	O3—C14—H14A	109.5
C3—C4—N3	120.26 (16)	O3—C14—H14B	109.5
C5—C4—N3	118.46 (17)	H14A—C14—H14B	109.5
C6—C5—C4	121.96 (18)	O3—C14—H14C	109.5
C6—C5—H5	119.0	H14A—C14—H14C	109.5
C4—C5—H5	119.0	H14B—C14—H14C	109.5
C5—C6—C7	117.02 (18)	N2—N1—C7	112.33 (17)
C5—C6—H6	121.5	N2—N1—H1N	118.9
C7—C6—H6	121.5	C7—N1—H1N	128.8
N1—C7—C6	131.46 (18)	C1—N2—N1	105.43 (17)
N1—C7—C2	106.32 (17)	C4—N3—S1	119.08 (13)
C6—C7—C2	122.21 (17)	C4—N3—H3N	114.7
C9—C8—C13	119.8 (2)	S1—N3—H3N	109.3
C9—C8—S1	121.26 (17)	C11—O3—C14	118.8 (2)
C13—C8—S1	118.83 (17)	O1—S1—O2	119.10 (9)
C8—C9—C10	119.6 (2)	O1—S1—N3	107.47 (10)
C8—C9—H9	120.2	O2—S1—N3	105.32 (9)
C10—C9—H9	120.2	O1—S1—C8	107.36 (10)
C11—C10—C9	119.7 (2)	O2—S1—C8	109.09 (10)
C11—C10—H10	120.1	N3—S1—C8	108.06 (9)
C9—C10—H10	120.1		

### Hydrogen-bond geometry (Å, º)

**Table:**

*D*—H···*A*	*D*—H	H···*A*	*D*···*A*	*D*—H···*A*
N1—H1*N*···O1^i^	0.90	2.07	2.956 (2)	168
N3—H3*N*···O2^ii^	0.77	2.23	2.998 (2)	172
C6—H6···O2^i^	0.93	2.52	3.381 (2)	155

Symmetry codes: (i) *x*+1, *y*, *z*; (ii) −*x*, −*y*, −*z*.
